# Treatment of Decay in Proximate Surfaces of Bicuspids and Molars

**Published:** 1896-09

**Authors:** Julius G. W. Werner

**Affiliations:** Boston, Mass.


ARTICLE X.

### TREATMENT OF DECAY IN PROXIMATE SURFACES OF BICUSPIDS AND MOLARS.

BY JULIUS G. W. WERNER, D. M. D., BOSTON, MASS.

For the average person the practice of dentistry need be no more tiresome nor detrimental to health than other professional occupations. When a dentist, working at the chair from six to eight hours a day, becomes feeble in health, pale and careworn before or about the time he reaches middle life, it must in a great degree be attributed to a constant neglect of laws governing health. Eight hours' work, eight hours devoted to recreation (and by recreation I mean physical and mental culture, counteracting injurious tendencies one finds in his daily occupation), and eight hours sleep and rest ought not to be considered a hard lot, even for a dentist.

I find my calling a source of pleasure and satisfaction that I should not expect to find in the general practice of medicine, or in any of its specialties, except dentistry. With our modern mechanical adaptations, improved instruments and the less pain-producing methods of operating, dentistry need not be very painful nor fatiguing to either patient or operator.

To us it should be a source of great pleasure and satisfaction that such a large percentage of the operations we are called on to perform are of a surgico-mechanical nature, whose *modus operandi* and prognosis are well understood, scientific and eminently satisfactory. With us it is surgery and mechanical art that stop decay and restore lost tissue and function. No pills concentrated or diluted *a la* Hahnemann; no mind-cure, Christian science, hypnotism, or the like, will ever cure or help in restoring decayed tooth-substance.

In filling of proximate surfaces of bicuspids and molars, and on the great importance of such decayed surfaces being restored to full or very full contour. Let us consider, then, that we have decay between two bicuspids involving the greater portion of the proximate surfaces, and where good judgment and favorable conditions call for a lasting or so-called "permanent" filling.

If such cavities are filled with cement, at the end of a year, or two at the longest, the fillings will have become worn sufficiently to need refilling. If they are not then refilled to a flush or full-contour condition, the proximate surfaces will either become unnaturally crowded together, or there will be a space left between the teeth, affording lodgment for food, which makes it uncomfortable, unclean and irritating to the gum. If such decayed proximate surfaces are filled with gutta-percha, a practically similar defective condition will occur in a short time, if filled with amalgam, we get discoloration of tooth-substance, oxidation of the filling-material, and weak edges, particularly at the cervical wall. Again, amalgam, as to looks and, according to the belief of many, as to health, is not a very desirable filling-material.

What must be considered then, on the whole, the most desirable and lasting filling for such cases? It is gold, soft, non-cohesive gold, with perhaps gold and tin at the cervical wall, and gold and platinum near the grinding surface. These filling-materials, from their malleability, can be so adapted to decayed and worn tooth-substance, and are so compatible with it, as to restore perfect contour, and, when skilfully used, will prevent decay for many years, in some cases forever. I have always been a thorough believer in gold, and am more strongly convinced to-day than ever of its merits as a filling material, when skilfully used, and when full contour, and even more than full contour, of the decayed tooth-substance is restored.

The merits and demerits of gold depend on the judgment and skill of the operator much more than on a so-called compatibility or incompatibility with tooth-substance. That all-hammered, all-cohesive-gold fillings, which only restore partial contour, should fail in anything but the very best tooth-substance need not surprise or discourage any one. It is very different when soft or non-cohesive gold, under matrix pressure, is thoroughly adapted to the cervical wall and all the edges of the cavity, and cohesive gold is used only near the grinding surface, and the whole filling is a thoroughly condensed and a well-rounded-out full contour operation.

To go still further, when soft, non-cohesive gold in conjunction with tin-foil is firmly packed against the cervical wall, and eight-tenths of the cavity is filled with soft or non-cohesive gold and the remaining two-tenths with cohesive gold and platinum, we then have what has proved, in my experience, an eminently satisfactory and decay-preventing combination. Such fillings will show, after a while, in many cases a slight oxidation of the tin and gold portion at the cervical wall, but no discoloration of tooth-substance, and give a condensed, hard grinding surface that in years after will show little if any wear and, I may say, little or no decay. But you may say that such operations will involve an amount of time and pain to the patient that will make it difficult to apply it in practice as a general rule. To this I reply an emphatic "No." To the one skilled in adjusting a properly shaped steel matrix, such operations are reduced to simplicity for the operator and comparative comfort to the patient.

Let us take a case in question: We have two extensively decayed proximate surfaces of either bicuspids or molars. If they have been filled with either cement, gutta-percha or amalgam, and their worn or defective surfaces have allowed the teeth to come together, the opposite of full contour, spread them apart sufficiently to gain the necessary space. This done, the teeth should be allowed to rest from two weeks to two months, as may be deemed most advisable. By so doing the teeth become firm and rested in their new and proper place, and the gum is pressed out and away from the decayed surfaces, giving space and accessibility to all portions of the tooth to be operated on. This spreading apart of the teeth and pressing away of the gum can be achieved in various ways—for instance, with gutta-percha, cotton, waxed linen tape, the Perry separator, or all combined. If they are cavities of decay with no fillings, with soft and sensitive dentine, disinfect the cavities with campho-phenique, or oil of cloves, and fill temporarily with cement, in whole or in part, excavating little or none, and proceed with the separation to acquire the desired space. Cases treated preliminarily in this way are, when finally and permanently operated on with gold and the matrix, in a much improved condition, and the patient experiences less pain and annoyance, the tooth not being sore to the necessary pressure.

To adjust a steel matrix, hold such firmly in its proper position with a wooden wedge at the cervix, gutta-percha being packed all around it, and, if necessary to give steadiness or to gain more space, to apply a Perry separator, is to the experienced but the work of a few moments. Nor is a properly shaped steel matrix firmly and accurately adjusted, painful to the patient, but, on the contrary, it steadies the tooth to be operated on and gives to the patient a feeling of support and comfort. Having the cavity properly excavated and shaped, and the matrix firmly adjusted, we began to fill to full contour.
A much-favored method with me is to fill a small portion of the cavity, that of the cervical wall, with soft or non-cohesive gold and tin, in proportion of one sheet of No. 4 gold to one-half sheet of No. 4 tin-foil, folded into suitable strips, with the gold on the outside and the tin inside. I believe soft gold and tin at the cervical wall to be a better decay-preventive filling than gold alone, and years of practice in this, both with matrix and without, have confirmed this belief. The slight oxidation of the tin seems a direct benefit, and has no special disadvantage, for we get no tooth-discoloration from it, as in the case when amalgam is used.

The non-cohesive gold and tin-foil at the cervical wall and all that postion which is of soft or non cohesive gold should be condensed with hand-pressure. The last two-tenths of the filling, the portion which comes toward the articulating or occluding surface, is made of cohesive gold, or, what I like better yet, of gold and platinum, and it should be annealed and hammered well into position.

We have, then, at the cervical wall soft or non-cohesive gold and tin, then the greater portion of the filling of soft gold, and the remainder of gold and platinum. All but the last portion of the filling is made with hand-pressure, the whole being so shaped with the matrix as to make it a well-rounded, solid-fitting, full-contoured filling.

What do I believe, then, to be the essentials of success in treating decayed surfaces of bicuspids and molars? First, the matrix; second, soft or non-cohesive gold and tin at the cervical wall, and gold and platinum at the cervical wall, and gold and platinum at the occluding surface; and, third, the whole filling to be solidly and thoroughly condensed against the walls of the tooth and matrix, making it well rounded out to full contour, at or near the grinding surface, with a free space near the neck of the tooth and the gum. When two proximal surfaces are filled in this way, nothing but gold touches, and the tooth edges are far apart.

In cases where it has been necessary to do considerable separating of the teeth, and where the gum had to be pressed out and away from the margins of cavities, the gum will grow down and fill nearly all the clear space that always should be near the neck of the teeth.

In properly shaped full-contour work each act of deglutition causes enough suction around and about the cervical wall, the neck of the tooth and the edges of the filling to keep the secretions changed, and in this way helps materially toward lessening redecay.

The one who has experienced the discomfort and annoyance, amounting at times to actual painfulness, resulting from spaces between proximate surfaces of bicuspids and molars, where food crowds in, irritating and inflaming the gum, can appreciate the comfort, satisfaction, healthfulness and cleanliness that exist where full contour keeps out food and protects the gum.

In my estimation no words are strong enough to condemn the semi-barbaric method of filling teeth apart, leaving so-called self-cleaning spaces, which really are and should be called "self-filling" spaces, or of leaving any space between, or any flat approaching fillings. Such are unnatural, unscientific and unclean. Non-comprehension of Nature's design, unskilfulness and incompetency are accountable for such methods of separating.

How different when, by a thorough understanding of what is needed and a love for our calling and duty, a delicate and firm hand becomes skilled enough to restore such decayed proximate surfaces to full contour, comfort, beauty, usefulness and permanence.—*International Dental Jonrnal.*

